# Toward Generalized Solution-State ^1^H DNP NMR via Particle-Mediated Cross-Relaxation

**DOI:** 10.1021/acs.jpclett.5c01398

**Published:** 2025-06-20

**Authors:** Sungsool Wi, Angeliki Giannouli, Korin Butbul, Jenica Lumata, Thierry Dubroca, Faith Scott, Zachary Dowdell, Robert W. Schurko, Hans Van Tol, Lucio Frydman

**Affiliations:** † 189689 National High Magnetic Field Laboratory, Tallahassee, Florida 32304, United States; ‡ Department of Chemical and Biological Physics, 34976 Weizmann Institute of Science, 7610001 Rehovot, Israel; § Department of Chemistry and Biochemistry, 7823 Florida State University, Tallahassee, Florida 32306, United States

## Abstract

This study discusses a potential route for enhancing ^1^H NMR signals in the liquid phase at high magnetic fields for samples on the 100 μL volume scale using dynamic nuclear polarization (DNP). The approach involves dispersing an inert powder that is both rich in protons and capable of undergoing DNP with good efficiency at noncryogenic temperatures, and letting the solid ^1^H polarization thus enhanced pass from the dispersed particles onto the surrounding liquid via spontaneous cross relaxation effects. To this end, BDPA-doped polystyrene (PS) particles in the μm range were suspended in 30 μL of heptane, loaded into 3.2 mm sapphire rotors, and spun at ≈500 Hz for homogeneity purposes in a 14.1 T magnet. Irradiation with ≈13 W at 395 GHz while maintaining temperature in the 185–220 K range thus enhanced the PS proton polarization ≈12-fold within ≈2 s; after ca. 6 s of irradiation, this resulted in ca. 3-fold enhancements of the heptane proton resonances, while preserving their ≤2 Hz line widths. The conditions over which such particle-mediated transfer occurs were explored over a range of sample composition, deuteration and molecular weight; best results were obtained when polarizing a ball-milled powder made of deuterated-PS/PS/BDPA = 86.4/9.6/4.0 suspended on perdeuterated heptane-*d*_16_. While the solution-state enhancements provided by this approach are still relatively modest, its generality could open new avenues in DNP-enhanced ^1^H NMR that do not sacrifice on the volumes, on the high-resolution conditions, or on the multiscan averaging that is customary in contemporary applications.

## Graphical abstract

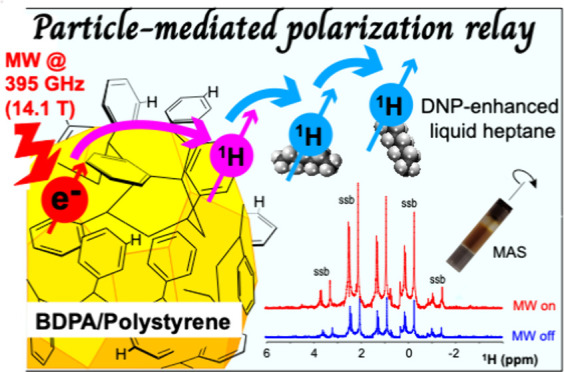

Although NMR is a powerful tool for characterizing molecular structure and dynamics,^1–4^ it suffers from poor sensitivity. This reflects the low polarization levels reached by nuclear spins at ambient temperature, even when placed in high magnetic fields.^1^ By transferring order from more highly polarized, comixed electrons, dynamic nuclear polarization (DNP) can dramatically boost NMR’s sensitivity.^5–7^ The past decade has seen much of this enhancement potential enabled for solid-state NMR, in experiments combining Gyrotron-based high-power microwave (μwave) irradiation, with the cryogenic conditions needed for enabling a saturation of the electronic spin.^8–11^ Early successes at low fields (*B*_0_ < 1 T)^12–18^ also bode well for the potential of solution-state DNP NMR; this, however, has yet to reach maturity due to the nearly negligible spectral densities needed for a generic electron → nuclear cross-relaxation-driven transfer, at the higher *B*_0_ fields of interest in analytical NMR. Foremost among the alternatives that have been stimulated by liquid-state NMR’s needs stands dissolution DNP, which bypasses liquid-imposed limitations by performing the polarizing process at cryogenic (≈1 K) temperatures and then suddenly melting and flushing the sample pellet as a solution for room-temperature high field observations.^16^ This has launched a revolution in metabolic imaging,^19–21^ yet the relatively high dilution that this method requires, its limitation to sites with slow-relaxation and nuclides with low-γ values, and its “single-shot” nature constrain its analytical uses.^22–25^ Further efforts have included low/high field sample shuttling experiments,^14,26–28^ rapid sample melting followed by subsequent refreezing and repolarization,^29–32^ and proposals based on biradicals.^33,34^ *In situ* applications based on the Overhauser-Effect DNP (OE DNP) effect have also been demonstrated at high fields and on relatively large samples, based on scalar electron/nuclear interactions.^27,35–38^ At high magnetic fields, however, OE DNP enhancements are critically dependent on the presence of substantial Fermi contacts, and hence limited to certain electronic environments; in particular, scalar-based OE DNP is of negligible use in the most common of all analytical NMR experiments: 1D ^1^H NMR.^38–41^ Closer to the spirit driving this study, OE DNP demonstrations carried out entirely in the liquid state at high magnetic fields based on high-power, high-efficiency microwave technologies have been reported,^42–45^ based on ubiquitous electron–proton dipolar effects.^46–48^ While these approaches could provide a general means to enhance protons in any molecule, these high-frequency, high-intensity microwave field experiments are compatible only with small, nanoliter-sized sample volumes. Water-oriented proposals that are somewhat related to the present study have been recently demonstrated, whereby hyperpolarization achieved on optically excited nanocrystals is relayed to the H_2_O protons, via low field microwave-based manipulations, followed by solid → solution cross-relaxation among protons.^49,50^

Inspired by these developments, the present study explores a complementary, potentially generic approach to enhance solution state ^1^H NMR at high fields, while targeting organic samples in the tens of μL. Dielectric losses limit such large-sample, high-field approaches to minimally absorbing solvents; while this leaves high-field water studies outside its realm, it should still be possible to target a considerable part of the organic/pharmaceutical NMR world with this kind of experiments. Making an organic-oriented DNP approach general would also demand a reliance on dipolar DNP effects, but as mentioned, these have negligible ^1^H efficiencies in high-field NMR. To overcome this pitfall, we propose an experiment that targets a solid powder that will still undergo an efficient, solid-like DNP enhancement, even while dispersed in an organic solvent. Further, we propose choosing these particles to be sufficiently small in size and sufficiently rich in protons, to enable their DNP-enhanced ^1^H polarizations to spontaneously spread into the liquid phase via ^1^H–^1^H cross relaxation; this in turn will have to be aided by solvent diffusivity in-and-out of the polarizing particles’ surfaces, and by relatively long relaxation times *T*_1_ so that a large number of molecules in the solution’s bulk can come into contact with the polarizing particles.^50^ The emerging Particle-Mediated DNP (PM-DNP, Scheme 1) proposal that is hereby discussed therefore not only has points in common with the aforementioned work by Prisner, Yanai et al.^45,49,50^ but also has clear differences. In particular, the polarizing particles in PM-DNP will simultaneously have to fulfill two seemingly irreconcilable tasks: undergo high-field electron → nuclear polarization as if they were a solid and be dispersed in a medium with which they can undergo efficient cross-relaxation as if they were in a liquid. A number of solute/solvent systems could be used to achieve these dual roles; in the present study, attention centered on polystyrene (PS) as prototypical example. PS is available on a large range of micro- and nanoparticulate sizes and is often used as DNP calibration sample thanks to its ability to undergo efficient Overhauser and/or Solid Effect (OE, SE) enhancements over a wide range of temperatures and of magnetic fields.^8,51–53^ In particular, when doped with 1,3-bis­(diphenylene)-2-phenylallyl (BDPA), it has been shown^53,54^ that a certain degree of deuteration helps in enhancing the efficiency of PS’s DNP enhancement, including at high temperatures. For the present study, mixtures of conventional and deuterated polystyrene (dPS, with all the eight hydrogens in the monomers replaced by ^2^H) were thus assayed, until reaching a suitable combination; also, a variety of solvents on which to test the transfer of DNP-enhanced polarization to solutions was assayed. In the end heptane, a solvent with low dielectric losses that will not dissolve PS nor BDPA, was identified as a suitable medium to assess these proposals. Also, in this case, heptane deuteration was found beneficial, presumably because it lengthened the solvent’s *T*_1_ and thereby enabled the DNP-derived buildup to proceed further throughout the full sample volume.

**Scheme 1:**
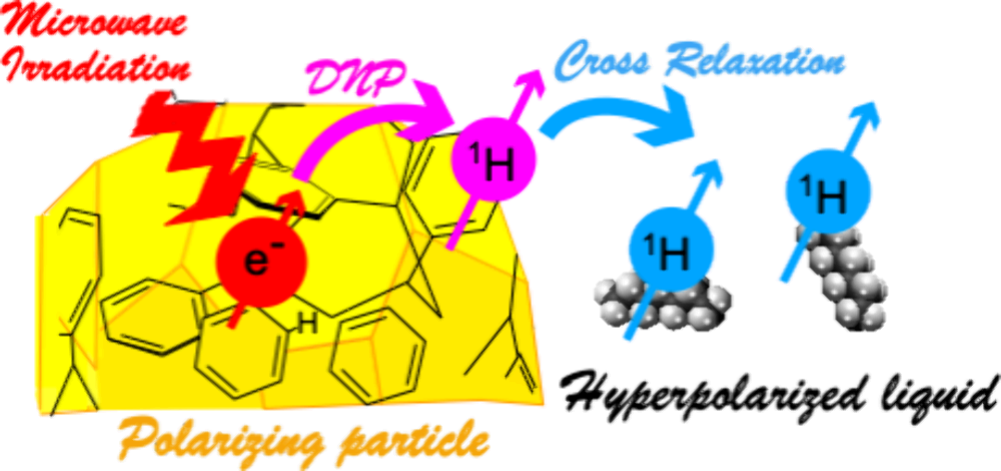
Representation of the PM-DNP Approach Adopted in This Study, to Polarize a Liquid with the Aid of Dispersed Particles Undergoing Efficient Microwave-Driven OE DNP in the Solid State

Figure 1 illustrates representative solid-state characteristics of the BDPA-doped PS samples analyzed in this study, recorded at 230 K while undergoing magic angle spinning (MAS) at ≈6 kHz (see Experimental Section for further details). The ^1^H DNP field sweep profiles arising from the powders reveal a strong OE DNP enhancement at a field of 14.107 T, corresponding to a ^1^H resonance frequency of 600.65 MHz and an electron Larmor frequency of 395.417 GHz, the latter being dictated by our Gyrotron’s fixed output frequency. Further sweeps of the magnetic field evidence a second, SE-enhanced DNP effect occurring at *B*_0_ = 14.085 T—equivalent to an electron frequency shift from the main OE event by 599.72 MHz, corresponding in turn to a shift by the ^1^H Larmor frequency. These plots were recorded for samples where the dPS had high (431 kDa) and low (2.2 kDa) average molecular weights (HM_W_, LM_W_), while keeping the weight ratio of the three components in the mixture—dPS/PS/BDPA—constant at 86.4/9.6/4.0. The HM_W_ sample exhibited a larger ^1^H DNP enhancement (ε = 19.3 over thermal at the OE condition) than the LM_W_ sample counterpart (ε = 11.4 at the OE condition). Figure 1 panels b and c show the μwave on/off signal build-up curves for the ^1^Hs in these samples, as measured at the optimal OE DNP (600.65 MHz ^1^H Larmor) position. For the microwave-off case, both samples exhibit similar 1.4–1.5 s build-up times. When the microwave was turned on, the HM_W_ dPS sample showed a build-up time that was approximately twice as long as that of the LM_W_ one and a concomitantly higher enhancement level. These differences likely reflect the different environments felt by the BDPA dopant in the PS matrix, where chain mobility and thereby electronic relaxation properties will be influenced by molecular weight. Figure 1 panels d and e complement these solid NMR results with ^1^H MAS and ^1^H–^13^C CPMAS NMR spectra recorded for the LM_W_ sample, showing a similar ε ≈ 11 OE DNP enhancement for both spectra.

**Figure 1:**
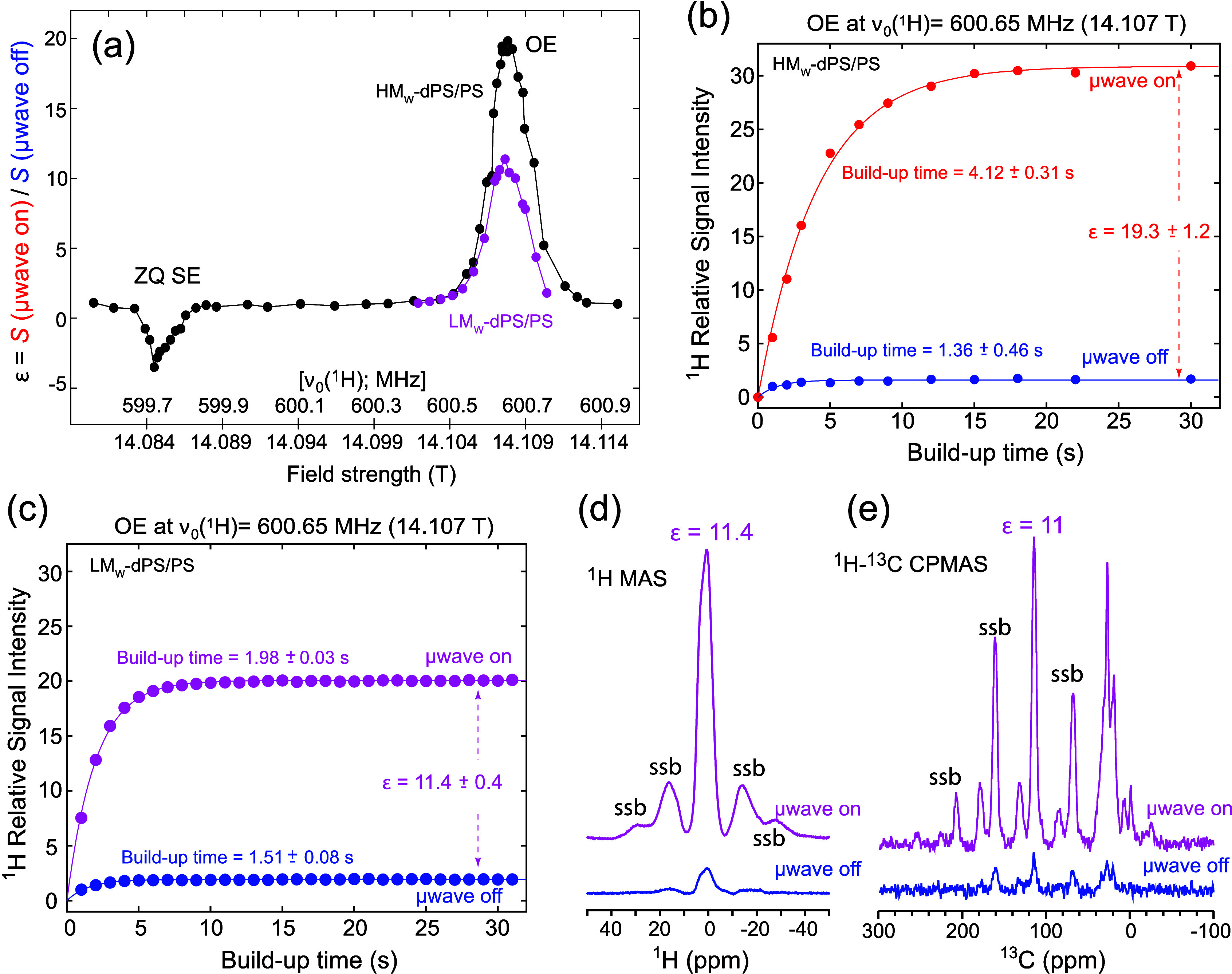
(a) DNP field sweep profiles of BDPA-doped dPS/PS solid samples containing high molecular weight (HM_W_) (431 kDa; black) and low molecular weight (LM_W_) (2.23 kDa; pink) dPS. Both samples comixed with nondeuterated PS (MW = 280 kDa) at a dPS/PS weight ratio of 90/10, with w/w 4% BDPA doped into the polymer matrix. Indicated are the positions of the Overhauser Effect (OE) and Zero-Quantum Solid Effect (ZQ SE) observed at the indicated field strengths, upon sweeping the magnetic field (and thereby the ^1^H Larmor frequency, which changed as indicated) while keeping the electron irradiation frequency constant. (b, c) Microwave on/off build-up curves observed at the central OE position for the HM_W_ and LM_W_ dPS samples, respectively. (d, e) Corresponding ^1^H MAS spectra and ^13^C CPMAS NMR spectra arising for the LM_W_ dPS sample, in the presence and absence of μwave irradiation at the OE condition. Data in (a−c) arose from ^1^H NMR spectra (e.g., d) recorded following a saturation recovery sequence, [90° (^1^H) - 1 ms]_n_ - τ_*buildup*_ (*n* = 50), with 13 W of μwave irradiation alternated between on and off states during the signal build-up time τ_*buildup*_. A 90° - τ_1_ - 180° - τ_2_ echo sequence was applied after τ_*buildup*_, with τ_1_ = 250 μs and τ_2_ = 0 μs; for ensuring thermal stability, experiments included a 60–80 s recycling delay. In (e), a 1 ms long CP block was included after τ_*buildup*_ to transfer ^1^H magnetization to ^13^C, with ν_1_(^13^C) = 50 kHz and ν_1_(^1^H) = 55 kHz (using a 70–110% ramp). Sixteen scans were coadded with SPINAL-64 ^1^H decoupling (^1^H rf ≈100 kHz).^55^ Throughout all experiments, samples were spun at approximately 6 kHz, and the temperature was maintained at 230 K. ssb denotes spinning sidebands.

As our aim is to enhance ^1^H solution-state NMR by transferring the amplified ^1^H polarization arising in the DNP-enhanced dPS/PS particles, these were ball-milled into fine powders before dispersing them into a solution. This should help leverage the intermolecular ^1^H–^1^H cross relaxation between the surface ^1^Hs of fine dPS/PS particles and the solvent. Hexane and heptane were tested as the latter, since these do not dissolve BDPA or PS, and are minimal absorbers of microwaves. In the end, the focus was on heptane, as its higher boiling point (99 °C) allowed it to better withstand microwave heating. Addition trial-and-error tests (data not shown) led us to adopt fully perdeuterated heptane-*d*_16_ (99 atom % D) as preferred medium, as it led to the highest enhancements when focused on its residual ^1^H nuclei.

With this as background, Figure 2a shows EPR spectra recorded on the microsized particles of BDPA-doped dPS/PS employing a LM_W_ sample. Two samples are here examined: a “wet precipitate” remaining after the PS particles are suspended in hexane or heptane and then settle in the bottom of an Eppendorf; and a more translucent “supernatant” containing particles that remain in suspension (see Supporting Information Section 1 for further description of these samples). These spectra were measured at 240 GHz, the closest that in EPR frequency could be accessed to the DNP NMR experiment, as well as at X-band (9.7 GHz). The high field EPR spectra recorded for the wet precipitate and for the supernatant present line shapes which are similar among themselves, as well as to the solid powder. By contrast, the X-band spectra of the supernatant, and to some extent also of the “wet precipitate” samples, display a fine structure that we attribute to a now-resolved e^–^-^1^H hyperfine interaction. The supernatant line shape also indicates that the dispersed particles are tumbling fast enough to average out the anisotropic hyperfine interaction, whereas the slower tumbling of “wet” sample has a broadening reflecting incomplete motional averaging. EasySpin simulations^56^ were performed to estimate rotational correlation times from these data (Supporting Information, Section 2): a radical correlation time of 2 × 10^–7^ s can account for both the X-band and 240 GHz supernatant sample spectra, while a slower correlation time (≥3 × 10^–7^ s) is required to fit the EPR spectrum of the wet precipitate. Assuming radicals that are fixed to the PS environs, these correlation time estimates predict particle sizes in the order of 1 μm. Dynamic light scattering experiments performed on similar samples (Supporting Information, Section 3) indicate larger particle diameters (≈4 μm); this probably reflects the respective biases of the EPR and DLS measurements to assess sizes, according to their different sensitivities.

**Figure 2:**
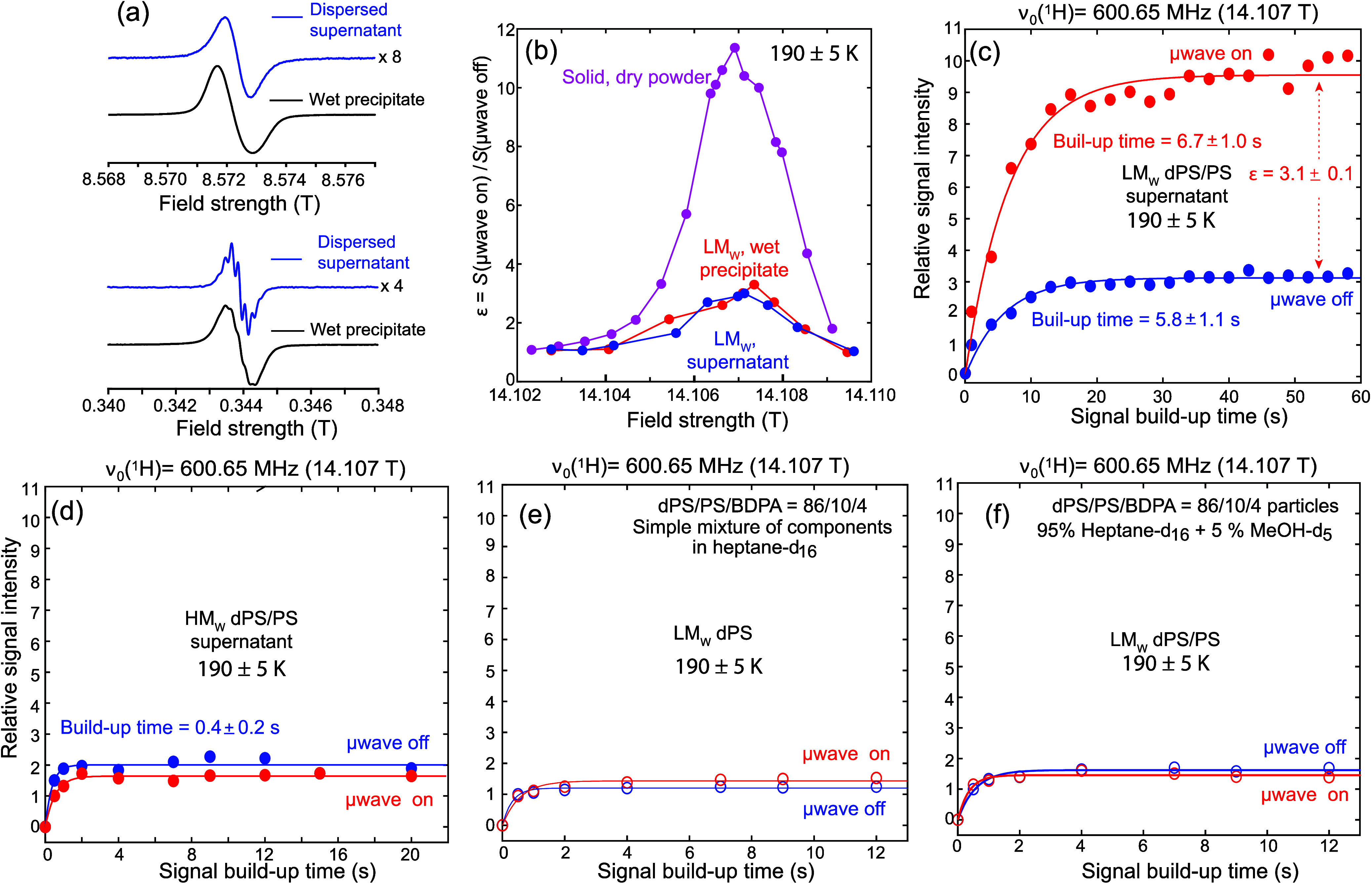
(a) EPR spectra of a LM_W_ dPS sample (dPS/PS/BDPA = 86.4/9.6/3.8 in weight ratio) measured at room temperature at 240 and 9.7 (X-band) GHz, in wet precipitates (black) and supernatant (blue) of the fine powders dispersed in *n*-hexane. (b) PM-DNP enhancement profiles obtained for the LM_W_ sample around the OE position for both supernatant (blue) and wet precipitates (red) in heptane-*d*_16_, as well as for the LM_W_ dPS powder (pink). Blue and red profiles were generated by plotting the intensity ratios measured for the residual ^1^Hs in liquid heptane-*d*_16_ in the presence or absence of microwaves, while varying the magnetic field (and ^1^H Larmor frequency) around the central OE position. Build-up time = 15 s; *d*_1_ = 60 s. (c) ^1^H DNP build-up curves obtained for the residual ^1^Hs in liquid heptane-*d*_16_ as driven by dispersed BDPA-doped dPS/PS LM_W_ particles, observed by toggling the microwave irradiation on (red) and off (blue) over a duration of up to 60 s (*d*_1_ = 80 s, 190 K, MAS rate ν_r_ = 700 Hz). (d)–(f) Measurements conducted under the same conditions as in (c), but for the indicated samples. No OE DNP enhancement was observed in any of these cases for the ^1^H spectrum of the residual solution-phase heptane.

Figure 2b presents field sweep profiles recorded around the OE DNP position for the solution NMR signals emanating from supernatant (blue) and wet precipitate (red) samples obtained from the LM_W_ PS preparation. These profiles reflect the intensity ratios of ^1^H NMR spectra measured at ∼190 K with μwave irradiation on and off, for the residual ^1^Hs in the liquid heptane-*d*_16_ used to suspend the PS particles. Although the relative DNP enhancements of these integrated ^1^H liquid spectra are smaller than the enhancements originating in the dPS/PS/BDPA powder itself (pink), the frequency-dependent features of their sweep profiles coincide with those of the solid sample. This suggests that it is the dPS/PS/BDPA particles dispersed in the heptane that are generating the DNP-driven enhancement of the latter’s protons and that no significant μwave-derived thermal heating effects, which should be independent of the minor field changes here involved, are biasing these experiments. Figure 2c shows the ^1^H particle-mediated DNP build-up curve observed for the heptane protons in the supernatant LM_W_ dPS/PS/BDPA sample, taken at the optimal OE position (600.65 MHz) and at a (calibrated) sample temperature of ≈190 K, while spinning the rotor at ν_r_ = 700 Hz (see Experimental Section and Supporting Information Section 4 for further technical details). The observed μwave-driven enhancement ε = 3.1 ± 0.1 of the heptane over its μwave-off counterpart, while ca. one-quarter of the value observed for the dPS/PS/BDPA sample in the solid state, is quite clear. The build-up time of this liquid-state enhancement is also longer than that of the solid dPS/PS/BDPA protons: 5.8 s vs 1.4 s. This is all consistent with the solid-DNP → liquid cross-relaxation mechanism that had been hypothesized. The precise value of the enhancement observed in the heptane depended somewhat on the exact composition of this LM_W_ dPS/PS/BDPA mix, but in general was maximum when PS was present in ca. 10% (Supporting Information, Section 5).

As illustrated in the Figure 2’s remaining panels, no similar enhancements were detected for other assayed formulations. The HM_W_ PS sample, which gave a better enhancement than the LM_W_ counterpart in the solid state, did not enhance heptane in the supernatant phase at 190 K (Figure 2d). We speculate that this could be due to an inefficient cross-relaxation between the HM_W_ solid and the liquid protons, and/or due to the fact that the ^1^H *T*_1_ times in this liquid were too short for supporting a substantial cross-relaxation-derived buildup: indeed, the theoretical model further developed below would predict negligible PM-DNP enhancements for liquids that like this one, have *T*_1_ ≈ 0.5 s. Also important, no DNP enhancement was observed when other formulations containing LM_W_ dPS, PS and BDPA in a state that did not involve the suspended doped particles were assayed: Controls involving a mixture of dPS, PS and BDPA independently dissolved in heptane-*d*_16_ showed no effects upon μwave irradiation (Figure 2e), neither did a mixture of the particles suspended in heptane with a small percentage of methanol, which is a known PS dispersant leading to a “jelly-like” state (Figure 2f). This confirms a need for dispersed PS particles that are not dissolved in the solvent but can still interact intimately with it, perhaps also through a swelling of the polymer matrix, for the solid → solution chain of enhancing events to take place. The absence of a direct OE DNP effect on these samples also confirms that the enhancements observed are not artifacts associated with a heating of the sample, as driven by absorption of the μwave radiation by the solvent or radical.

Figure 3 panels a and b present examples of the ^1^H NMR spectra that gave the origin to the data in Figure 2. These spectra were recorded for the heptane in the supernatant samples, for μwave on and off conditions at 190 and 200 K. Being mostly deuterated and therefore devoid from homonuclear J-couplings, these spectra should exhibit sharp singlets at 0.86 (single methyl peak) and 1.26–1.27 ppm (up to three methylene peaks). Singlet-like peaks are indeed observed in the μwave on/off spectra, exhibiting narrow peak widths at half heights (1–2 Hz) as expected from an isotropic liquid. Supporting Section 6 illustrates additional examples of these PM-DNP enhanced ^1^H NMR experiments but for different polymer/radical compositions. A most evident feature of all these spectra is the presence of strong spinning sidebands, that repeat heptane’s singlets structure at multiples of the MAS rate. These phenomena are not reflective of solid anisotropies, but rather of susceptibility and rf inhomogeneity issues associated with the probe that was used;^57,58^ they were not associated with any sample composition and were also observed in neat heptane (Figure 3c) or even in water samples (Supporting Information, Section 7). These sidebands eventually span away at rates >2–3 kHz and are thus not usually observed in solid state MAS DNP experiments carried out at these or higher spinning rates. However, in the present case, spinning the rotor containing the supernatant sample at rates exceeding a few 100 Hz led to centrifugation of the dispersed dPS/PS/BDPA particles and to elimination of the observed liquid-state DNP enhancements. On the other hand, static sample experiments led to broad, ca. 1 ppm line widths, depriving the resulting spectra from a liquid-like character (Supporting Information, Figure S7). Consequently, experiments had to be conducted at the slowest possible rotation rates, which while leading to excellent resolution, were also accompanied by these sideband artifacts.

**Figure 3:**
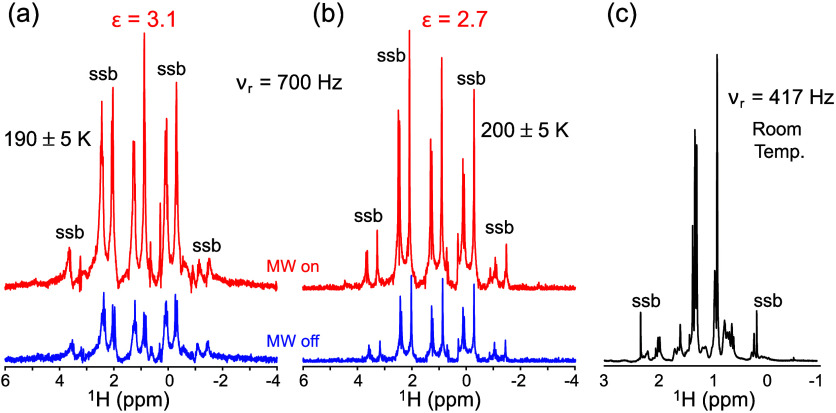
(a, b) Liquid-state ^1^H NMR spectra LM_W_ dPS/PS/BDPA (86.4/9.6/3.8 wt %) dispersed in heptane-*d*_16_ recorded with microwaves on (red) and off (blue), obtained at the indicated temperatures and conditions, and leading to the indicated PM-DNP enhancement factors. These enhancements arose after a signal build-up time of 15 s and a delay time (d1) of 80 s. Spectra were collected using the echo sequence described in Figure 1 (2 scans; τ_1_ = 250 us; τ_2_ = 0 us). (c) ^1^H NMR spectrum of heptane-*d*_16_ recorded at the indicated conditions. In all cases peak line widths were 1–2 Hz; ssb denotes spinning sidebands.

The PM-DNP experiment hereby presented was proposed based on a relayed BDPA → PS­(solid) → Heptane­(liquid) transfer process. As the characteristics of each individual polarization-transfer process in this chain can be independently measured, it should be in principle possible to check consistency between the overall enhancements and buildup time scales shown in Figures 2 and 3 and each step’s independent observables. Doing so requires, in turn, a theoretical framework linking the two sets of measurements. To this effect the cross-relaxation between the ^1^Hs of the PS (solid S) and those of the heptane (liquid L) were described as in terms of their enhancements ε over (in principle identical) thermal polarization values *S*_0_ and *L*_0_, *ε*_*S*_ = *S*/*S*_0_ and *ε*_*L*_ = *L*/*L*_0_. Starting from a DNP-enhanced set of Solomon equations linking these two phases, we can connect these enhancements as done in ref 50:

1 $$\frac{d\varepsilon_{S}}{dt}=B_{S}\left(\frac{P_{e}}{P_{S}}-\varepsilon_{s}\right)-R_{S}\left(\varepsilon_{S}-1\right)-\sigma_{SL}\left(\varepsilon_{L}-1\right)$$

2 $$\frac{d\varepsilon_{L}}{dt}=-R_{L}\left(\varepsilon_{L}-1\right)-\sigma_{LS}\left(\varepsilon_{S}-1\right)$$

Here *B*_*S*_ is the buildup rate constant for the ^1^H nucleus of the PS solid, brought about by a *P*_*e*_ electron radical polarization enhancing the *P*_*S*_ polarization of the PS ^1^Hs; *R*_*S*/*L*_ are the self-relaxation rates (1/*T*_1_) of the solid and liquid protons, and the σ’s denote the cross-relaxation rates between the two reservoirs. The ratio *P*_*e*_/*P*_*S*_ = *ε*_*S*_^0^ corresponds to the maximum efficiency that the μwave-driven OE DNP process will impart on the solid protons and is in essence the final ^1^H DNP solid-state polarization enhancement described experimentally in Figure 1c. Assuming for simplicity that this effect is much larger than the σ_*SL*_(*ε*_*S*_ – 1) losses due to polarization transfers to the solvent protons, the latter term can be disregarded and integration of eq 1 for the boundary condition *ε*_*S*_ = 0 at *t* = 0 yields

3 $$\varepsilon_{S}=\frac{B_{S}\varepsilon_{S}^{0}+R_{S}}{\left(B_{S}+R_{S}\right)}\left\{1-\exp\left[-\left(B_{ps}+R_{ps}\right)t\right]\right\}$$

Inserting this into eq 2 and integrating yields an analytical expression for the time-dependent enhancement being transferred to the liquid-state protons (see Supporting Information, Section 8):

4 $$\varepsilon_{L}\left(t\right)=\frac{a}{R_{L}}+\frac{c}{R_{L}-c}e^{-ct}+\left(1-\frac{a}{R_{L}}-\frac{b}{R_{L}-c}\right)e^{-R_{L}t}$$

where *a* = $R_{L}+\sigma_{LS}+\sigma_{SL}\left(\frac{B_{S}\varepsilon_{S}^{0}+R_{S}}{\left(B_{S}+R_{S}\right)}\right)$ , *b* = ${-\sigma }_{LS}\left(\frac{B_{S}\varepsilon_{S}^{0}+R_{S}}{\left(B_{S}+R_{S}\right)}\right)$ , and *c* = *B*_*S*_ + *R*_*S*_.

Several of the parameters involved in this equation, including the solids buildup rate and individual longitudinal relaxation rates, can and were measured in independent experiments: Figures 1 and 2 provide estimates for the relaxation and DNP buildup rates and maximal enhancements in the solid, Supporting Information Section 9 describes the saturation transfer difference (STD) measurements yielding the polymer → heptane-*d*_16_ cross-relaxation rates, and Supporting Information Section 10 presents the ancillary liquid state relaxation data. These yielded nearly identical 0.94 s relaxation times for the CDH and CD_2_H resonances in neat heptane-*d*_16_ and longer values 4.5 and 7.6 s, respectively, for these sites in the presence of the dPS/PS/BDPA polarizing particles. Figure 4 shows a fit of the ensuing liquid-state experimental PM-DNP enhancements to eq 4, plotted as a function of the μwave irradiation time. Assuming again a negligible σ_*LS*_ = 0 value, one can see that neither the average heptane-*d*_16_ *T*_1_ values in the presence (5.8 s, magenta line in Figure 4) nor in the absence (1 s, black line) of the polarizing particles provide a perfect fit for the buildup data. An intermediate *T*_1_ value of 2.6 s, however, quantitatively recapitulates the liquid buildup behavior. We believe that this reflects the fact that the heptane-*d*_16_ *T*_1_ values that matter in the calculation of the liquid-state DNP enhancement are derived from protons at the particle/liquid interface, whose relaxation properties are probably different from the ones measured by any bulk-based technique. When considering that, in addition, the *R*_*S*_ values measured probably also reflect the bulk of the PS rather than the relevant protons at the PS/heptane interface, that the PS enhancement values *ε*_*S*_^0^ were measured in the absence of the organic solvent, and that a number of approximations were taken (e.g., negligible polarization losses of the solid ^1^H polarization by the transfer to the liquid ^1^Hs, negligible liquid → solid polarization back-transfers), it is fair to say that the independent measurements and the aforementioned model reproduce satisfactorily the behavior in the liquid-state PM-DNP spectra.

**Figure 4:**
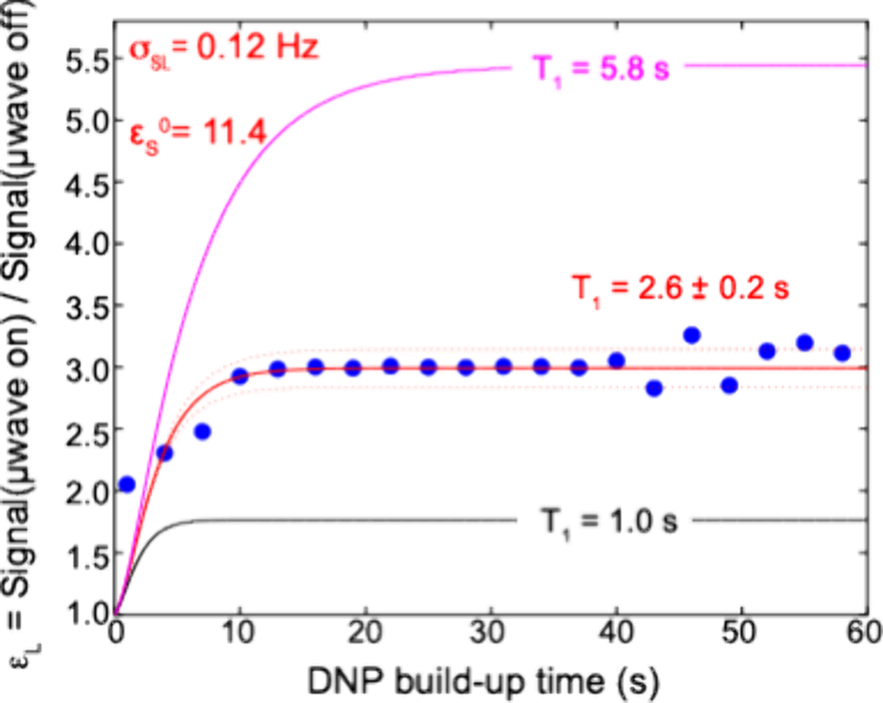
Fitting the experimental DNP-enhanced buildup data for heptane-*d*_16_, according to the multistep BDPA → PS­(solid) → Heptane­(liquid) polarization transfer mechanism presented in the text. A common (average) enhancement *ε*_*L*_ was taken for both methyl and methylene protons. Blue dots represent the experimentally obtained *ε*_*L*_ values calculated at each build-up time point; the solid red line represents the best-fit of eq 4 for σ_*SL*_ = 0.12 Hz, *ε*_*S*_^0^ = 11.4, *T*_1_(S) = 1.5 s, *B*_S_(PS) = 0.5 s^–1^, *T*_1_(heptane) = 2.6 s. Dashed red lines indicate error bounds for ± 0.2 s; magenta and black lines indicate the behaviors expected for the indicated *T*_1_(L) values.

A final feature worth discussing is the ca. 10-fold longer build-up time exhibited by the polarizing liquid in the low-*M*_w_ sample experiment in Figure 2b, vs the subsecond *T*_1_ build-up times evidenced by the nonpolarizing conditions in Figure 2c–f. To shed light on this, ^1^H *T*_1_ values were measured at 190 K on pure heptane-*d*_16_, and on a low-M_W_ sample optimized for the DNP experiment (heptane-*d*_16_ with dispersed dPS/PS/BDPA 86.5/9.6/3.9 wt % microparticles; see Supporting Information, Section 10). Once again, the liquid-state *T*_1_ relaxation times of these two samples differ significantly. In pure heptane-*d*_16_ the *T*_1_ values for both the methylene and methyl moieties were 0.94 ± 0.04 s—comparable to the build-up times in Figure 2c–f. By contrast, *T*_1_ values for the DNP-enhancing sample were substantially longer and akin to those measured in Figure 2b: 4.5 ± 0.4 and 7.6 ± 0.9 s for the methylene and methyl groups, respectively. These increases in *T*_1_ values induced by the dispersed microparticles, which might arise from viscosity changes but whose origin remains to be elucidated, facilitate in turn the cross-relaxation between ^1^H­(PS) on the particles’ surfaces and ^1^H­(heptane) in the liquid phase, and may be responsible for the differential DNP behaviors shown in Figure 2. Indeed, in all samples whose solution relaxation times *T*_1_ were short and similar to those observed in pure heptane-*d*_16_, no significant DNP effects built up by the relatively slow (σ_SL_ ≈ 0.12 Hz) solid → liquid cross-relaxation rate. Further reduction of the *T*_1_(L)*σ_SL_ product most likely are also responsible for the negligible PM-DNP enhancements observed when *T* ≥ 200 K (Supporting Information, Section 11).

In a search for the elusive goal of achieving ^1^H DNP enhancements on solution-state samples approaching the normal volumes used in analytical NMR, this study explored an indirect route based on e^–^ → solid-^1^Hs → liquid-^1^Hs particle-mediated DNP transfers. Doped PS was here chosen as the polarizing particles because of their ability to undergo DNP outside cryogenic regimes, which will usually be incompatible with solution state investigations. Organic solvents on the other hand can remain fluid over a range of temperatures, and hence, liquid-state organic NMR is not necessarily synonymous with room-temperature DNP NMR. As shown in the Supporting Information (Section 11), the solution NMR enhancements here achieved are in fact highly temperature dependent: Figure S11 shows that repeating the experiment illustrated in Figure 2c (190 K) using an identical sample and conditions other than for slightly lower (185 K) or higher (220 K) temperatures leads to noticeably higher or lower enhancements, respectively. A full characterization of these effects thus calls for careful measurements of the μwave on/off influence on sample temperature, which were here accounted for by the calibrations described in the Supporting Information, Section 4. In this respect, it was noticed that working at temperatures ≤180 K that are close to the heptane/PS freezing point risked changing the mobility of the heptane molecules, and hence their ^1^H NMR spectrum, by microwave irradiation—even when working off the OE condition. Temperatures at the melting point interface, however, were also accompanied by a merging of the multiple ^1^H heptane peaks into a single broader peak; these spectral changes were very evident and helped to avoid confounding factors.

The experiments here presented are still a distance away from practical analytical applications: the enhancements imparted by PM-DNP are still modest, the spinning sideband patterns are an annoyance, and enhancements of codissolved solutes remain to be tested. On the other hand, the solids DNP/liquid cross-relaxation mechanism appears general and open to several potential improvements. Extensive optimizations in the formulation, the hardware, and the conditions that can be assessed in this kind of experiment remain to be carried out. And while liquid-state high-field DNP investigations on samples in the 10–100 μL range are likely to be unfeasible in aqueous phases because of microwave penetration problems, it is worth remembering that most analytical NMR work still takes place in organic liquids. Hence, further progress in the PM-DNP area appears to be well worth the effort.

## Experimental Section

### Chemicals

Low molecular weight (LM_W_) deuterated polystyrene-*d*_8_ (dPS; *M*_W_ = 2.23 kDa, 98% D) and high molecular weight (HM_W_) dPS (*M*_W_ = 412 kDa) were purchased from Cambridge Isotope Laboratories and Polymer Source, Inc. (Quebec, Canada), respectively. Nondeuterated polystyrene (PS; *M*_W_ = 280 kDa) was obtained from Aldrich. Perdeuterated heptane-*d*_16_ (99 atom % D), methanol-*d*_5_ (99.8 atom % D), toluene, and BDPA (1,3-bis­[diphenylene]-2-phenylallyl) free radical (1:1 complex with benzene) were all purchased from Sigma-Aldrich and used as received. All solvents were deoxygenated by undergoing at least five freeze–degassing–purge (N_2_)–thaw cycles and were stored in a glovebox with a tight seal.

### Production of BDPA-Doped dPS/PS Powder Samples

60.0 mg of dPS, 6.7 mg of PS, and 2.8 mg of BDPA were placed in a 7.5 mL glass vial with a magnetic stirring bar. Approximately 200 μL of toluene was added. The target composition corresponds to doping about 4% BDPA into a polymer matrix consisting of 90% dPS and 10% PS, with the relative weight ratio of the components being dPS/PS/BDPA = 86.4/9.6/4.0. The mixture was stirred for about 2 h on a magnetic stirrer until it dissolved completely and homogeneously. The resulting solution was poured onto a glass plate to form polymer films, allowing toluene to evaporate at room temperature. To make the solid film more brittle and easier to grind into micropowders, the remaining toluene was removed using a vacuum oven without heating. Thin films of BDPA-doped dPS/PS samples recovered from glass plates after solvent evaporation were converted into microparticles by ball milling. Ball milling was performed to reduce the particle size using a Retsch Mixer Mill 400. The process utilized two 10 mL stainless steel jars, each containing six 3 mm stainless steel bearings, operated at a milling frequency of 30 Hz for a total of 1 h. Prior to milling, the jars with the samples and bearings were submerged in liquid nitrogen until vigorous boiling subsided (approximately 10–15 min). After every 10 min of milling, the jars were resubmerged in liquid nitrogen for around 5 min until boiling subsided, repeating this process until the 1 h milling time was complete. The low temperatures and use of numerous small bearings aimed to further minimize particle size.^59,60^ The ball-milled BDPA-doped dPS/PS powder samples were kept in a glovebox to remove any oxygen trapped in the solid matrix. Supporting Information describes how these samples were readied for the DNP NMR measurements.

### NMR Experiments

The 90° pulse lengths for ^1^H, ^13^C, and ^79^Br were 2.5 μs, 5 μs, and 5 μs, respectively. ^1^H NMR spectra were acquired by coadding two scans with an acquisition delay time of *d*_1_ = 60–80 s and a MAS rate ν_r_ = 200–700 Hz, while alternating the phases of both the 180° pulse and signal receiver between 0 and π. The echo delay times in the Hahn echo sequence were set to 250 μs before and 1 μs after the 180° pulse to preserve the early portion of the signal. For the ^1^H–^13^C CPMAS experiments carried out on the solid BDPA-doped dPS/PS powders, the parameters used were ν (^1^H) = 55 kHz and ν (^13^C) = 50 kHz for 1 ms for the CP mixing by employing a ramped (90%–110%) spin-lock pulses along the ^1^H channel. The ^1^H decoupling power used for the ^13^C detection was about 90 kHz while employing the SPINAL-64 sequence.^55^ The MAS rate used for the ^1^H–^13^C CPMAS experiment was about ν_r_ = 5 kHz. A 90° read pulse was applied after the saturation recovery sequence and a variable delay time to measure ^79^Br *T*_1_ for temperature calibration.^61^

### DNP Experiments

^1^H MAS DNP NMR spectra were acquired from the liquid heptane-*d*_16_ (ca. 99 % D) solution that is dispersed with BDPA-doped dPS/PS micropowders. These spectra were acquired using a widebore ^1^H 600 MHz Bruker Avance III spectrometer configured to a 395 GHz gyrotron microwave source and a Bruker 3.2 mm MAS ^1^H-X-Y triple-resonance probe. Approximately 30 μL of heptane-*d*_16_/dPS/PS/BDPA colloidal solution was filled into a 3.2 mm sapphire MAS rotor with a silicon rubber septum and a Vespel polyimide cap. The MAS spinning rate employed in the experiments was in the range of 200–700 Hz, while cooling the sample compartment to 185–200 K using a nitrogen gas stream evaporated from a liquid nitrogen tower. Approximately 13 W microwave power from the gyrotron source via the optic table was fed into the sample from the probe base. Microwave irradiation was applied to the sample rotor only during the signal build-up time that follows right after the signal saturation sequence, [90° (^1^H) pulse-1 ms delay]_*n*_ (*n* ≈ 50), while it was turned off during other parts of the sequence including the recycle delay time (*d*_1_). Microwave control was managed by opening and closing a shutter using TTL signals, precisely synchronized with the start and end of the signal build-up time in the pulse sequence. This ensured accurate on–off switching and optimized microwave usage by minimizing unnecessary irradiation during periods when it was not needed. The saturation recovery sequence was employed in all our sequences used in the experiments, including the ^1^H DNP NMR experiments, ^1^H-enhanced ^1^H–^13^C CPMAS experiments as well as ^79^Br *T*_1_ measurements (along ^79^Br) for temperature calibration.

All of the graphs in the main and supporting texts, along with the associated error ranges, were constructed and analyzed by using the curve fitting toolbox provided by the Matlab software.

## Supplementary Material



## Notes

Additional details on the NMR, EPR and dynamic light scattering measurements; self- and cross-relaxation rates; temperature and sample dependencies of the PM-DNP enhancement; nature of the spinning sidebands; full description of the PM-DNP mechanism and kinetic modeling (PDF)

## 4

Department of Chemistry, University of Crete, Heraklion 70013, Greece
