# Supplementary material for: Toward Generalized Solution-State 1H DNP NMR via Particle-Mediated Cross-Relaxation
Source: J Phys Chem Lett. 2025 Jun 20;16(25):6627–36. doi: 10.1021/acs.jpclett.5c01398 (PMC12207668; doi:10.1021/acs.jpclett.5c01398)
Supplement: Supplementary file 1 [file jz5c01398_si_001.pdf]

Supporting Information for

Towards generalized solution-state ^{1}H DNP NMR via particle-mediated cross-relaxation

Sungsool Wi,[1,*] Angeliki Giannouli,[2,4] Korin Butbul,[2] Jenica Lumata,[1] Thierry Dubroca,[1] Faith Scott,[1] Zachary Dowdell,[3] Robert W. Schurko,[1,3] Hans Van Tol,[1] and Lucio Frydman[1,2,*]

[1] National High Magnetic Field Laboratory, Tallahassee, Florida 32304, USA

[2] Department of Chemical and Biological Physics, Weizmann Institute of Science, 7610001 Rehovot, Israel

[3] Department of Chemistry and Biochemistry, Florida State University, Tallahassee, Florida 32306, USA

[4] Present address: Department of Chemistry, University of Crete, Heraklion 70013, Greece

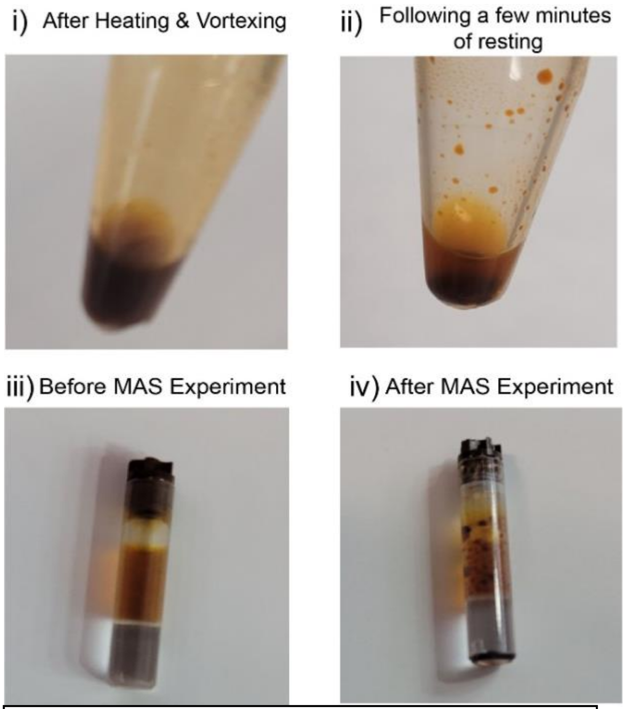

Figure S1. Images illustrating the preparation and sampling process of BDPA-doped dPS/PS particles dispersed in heptane-d$_{16}$ for MAS DNP experiments in the liquid state.

1. Preparation of heptane-d$_{16}$ solution with dispersed BDPA-doped dPS/PS powders

Approximately 15–20 mg of ball-milled solid powders of dPS/PS/BDPA, with a weight ratio of 86.4%/9.6%/4.0%, were placed in an Eppendorf tube inside the glovebox. Subsequently, about 50 µL of heptane-d$_{16}$ was added to the tube. After removing the tube from the glovebox, the colloidal solution for the experiment was prepared by briefly heating the mixture for 5–10 seconds using a heat gun. This was followed by vigorous shaking using a vortex mixer to ensure through mixing (i). As the mixture settled, it naturally separated into two distinct phases: the precipitate and the supernatant (ii). Approximately 30 µL of the supernatant, containing dispersed dPS/PS/BDPA particles (with a particle concentration of about 16 wt%), was carefully transferred into a 3.2 mm sapphire rotor inside the glovebox. A silicone rubber septum was placed on top before capping to prevent leakage during sample spinning for the DNP experiment (iii). Shown in (iv) is an image

of the sample rotor after MAS experiments, highlighting some precipitation from the initially clear, homogeneous solution.

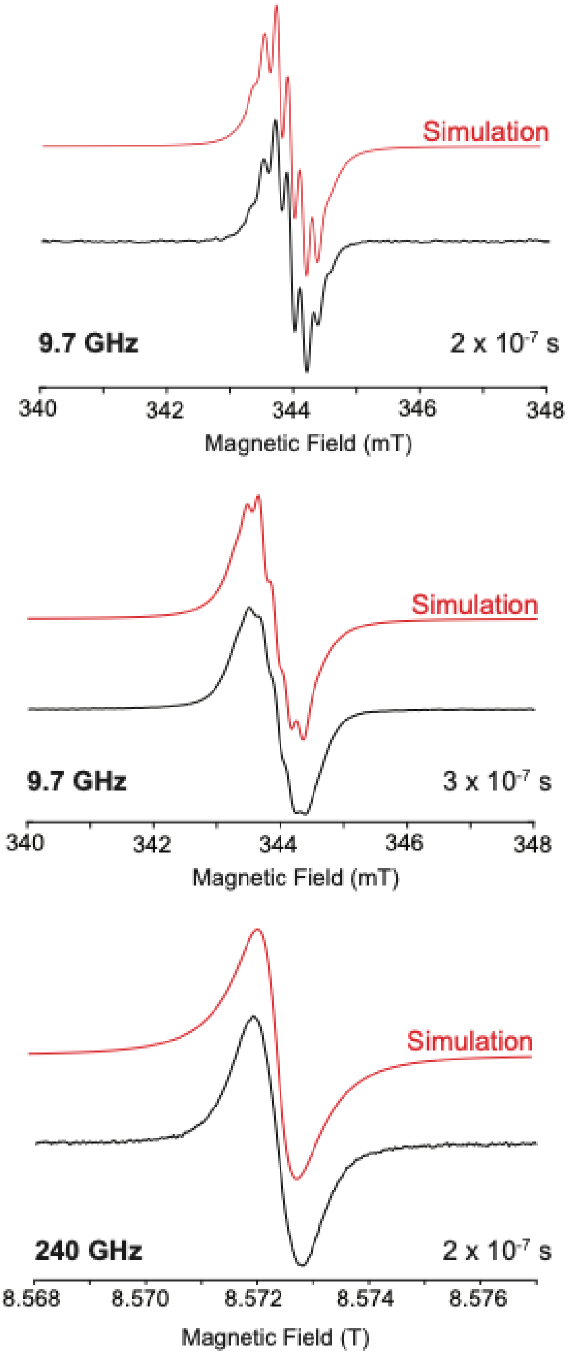

Figure S2. EasySpin-simulated EPR spectra with correlation times of 3×10^{-7} s and 2×10^{-7} s at 9.7 GHz and 240 GHz.

2. EPR experiments

High-field EPR at 240 GHz was conducted using a multi-frequency quasi-optical spectrometer, as described in our earlier publication,[1] with approximately 10 μL of sample placed in a sample holder without a resonator. The EPR spectra of the solid BDPA-doped dPS/PS powder and its dispersed heptane-d16 solution were measured using this sample holder. X-band EPR spectra at 9.7 GHz were measured using a desktop X-band EPR machine (EMXnano Bruker). The sapphire rotor, filled with either the heptane-d16 solution containing dispersed BDPA-doped dPS/PS powder or the wet precipitate intended for MAS DNP NMR experiments, was directly inserted into a 4 mm-diameter quartz tube for the EPR measurements.

EPR spectra measured at 240 GHz and 9.7 GHz at room temperature are presented in Figure 2a. The supernatant exhibits the hyperfine structure of the isotropic proton hyperfine component at 9.7 GHz, indicating that the beads are tumbling fast enough to average out the anisotropic hyperfine interaction. In contrast, the slower tumbling in the wet precipitate leads to broadening due to incomplete motional averaging. At 240 GHz, the symmetric line suggests that the tumbling is sufficiently fast to average out the g-anisotropy observed in the bead powder. To estimate the rotational correlation time for the e^{-}-^{1}H hyperfine interaction, simulations were performed using EasySpin, as shown in Figure S2. These simulations assumed isotropic values for BDPA’s 8 strongly-coupled protons as reported in Ref. 2; for protons in the alpha and gamma positions the anisotropic tensors were taken as 4 and 3 MHz respectively.[3] Orientations were taken arbitrarily, as there is most likely substantial stresses in the hyperfine values of the radical when immersed in the PS. A correlation time of 2×10^{-7} s accurately accounts for both the 9.7 GHz and 240 GHz spectra.

However, a slightly slower correlation time of 3×10^{-7} s is required to fit the EPR spectrum of the wet precipitate sample measured at 9.7 GHz (Figure 2 in the main text). Given the viscosity of the solvent $\eta = 427$ µPa·s at the tested temperature T = 290 K,[4] and assuming that the correlation times are described by $\tau_R = 8\pi\eta r^3/kT$, we predict a particle size of about 0.9 µm for the beads originating the EPR spectra.

3. Dynamic Light Scattering data

The particle size of the PS/BDPA sample was obtained by DLS measurement using a Malvern's Zetasizer Nano ZSP instrument and a quartz microcuvette with a capacity of 80 µL of sample. The equilibration time of the instrument was about 15 min and the temperature was set to 25 °C. The particle size was obtained from the output data form the software.

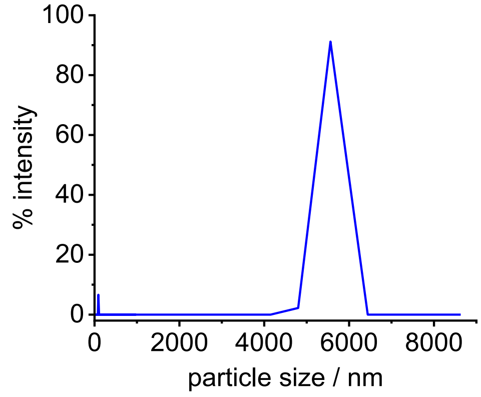

Figure S3. DLS data on PS beads with 5% BDPA The sample was prepared by dissolving PS (0.5 g) with BDPA (25 mg) in 1 mL toluene and thoroughly dried under vacuum. The created film was ball milled at 80 K (5 x 5min cycles) before being resuspended in 1 mL heptane and subjected to sonication before being measured with DLS at room temperature.

4. Temperature calibration

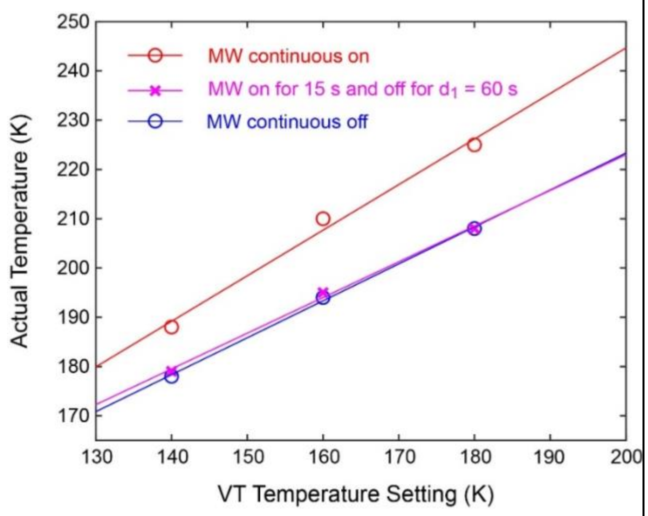

Figure S4. Temperature calibration experiment conducted on KBr, utilizing the T_1 relaxation of ^{79}Br. The saturation recovery T_1 measurement method was employed with three modes: continuous µwave on (red), µwave off (blue), and controlled µwave -on (pink). VT setting temperatures were adjusted to 140 K, 160 K, and 180 K. Across the temperature range tested, the actual sample temperature in the continuous µwave -on mode was approximately 10 to 20 K higher than in the µwave -off measurements. In contrast, the controlled MW-on mode, where the microwave was on only during the signal build-up time (15 s) and off during the rest of the sequence, including the acquisition delay time (d_1 = 60 s), resulted in temperatures nearly identical to those measured in the µwave-off mode. In each case, straight lines were obtained by least square fitting methods ($R^2 = 0.99 \sim 0.999$ in the 180 to 230 K range).

Figure S4 shows temperature calibrations carried out in our experiment using the known

temperature dependance of the T_1 time of ^{79}Br in solid KBr matrix.[5] The data for the blue and red circles were obtained by rotating the sample rotor at approximately 200 to 300 Hz (with bearing pressures of about 200–300 mB and drive pressures of about 10–20 mB), with the microwave turned off and on, respectively. As can be seen from these graphs, when the microwave is continuously irradiating, the sample temperature increases by about 10 to 20 degrees over the set temperature in the 180-230 K setting range. However, as shown by the pink (x-shaped) data, turning on the microwave for about 15 seconds and off for about 40 seconds, leads to a temperature increase of only 1 to 2 K. Although all experimental temperatures were based on this calibration, additional variations occurred in the settings of bearing and drive pressures during the actual experiments. Consequently, the error bound for the actual temperature calibrated was assumed at approximately ± 5 K. This margin arises from fluctuations in conditions, including variations in bearing and drive N_2 pressures of around 200–400 mB and 10–30 mB, respectively. These fluctuations are necessary to maintain a magic angle spinning (MAS) condition at a very slow MAS rate in the 200 to 700 Hz range, which can differ from sample to sample.

5. The effect of the dPS/PS ratio and the relative content of BDPA.

In principle, diluting the concentration of protons in the PS particles should be detrimental to the transfer of polarization from the particles to the solvent; at the same time, it is known that PS's ^1H

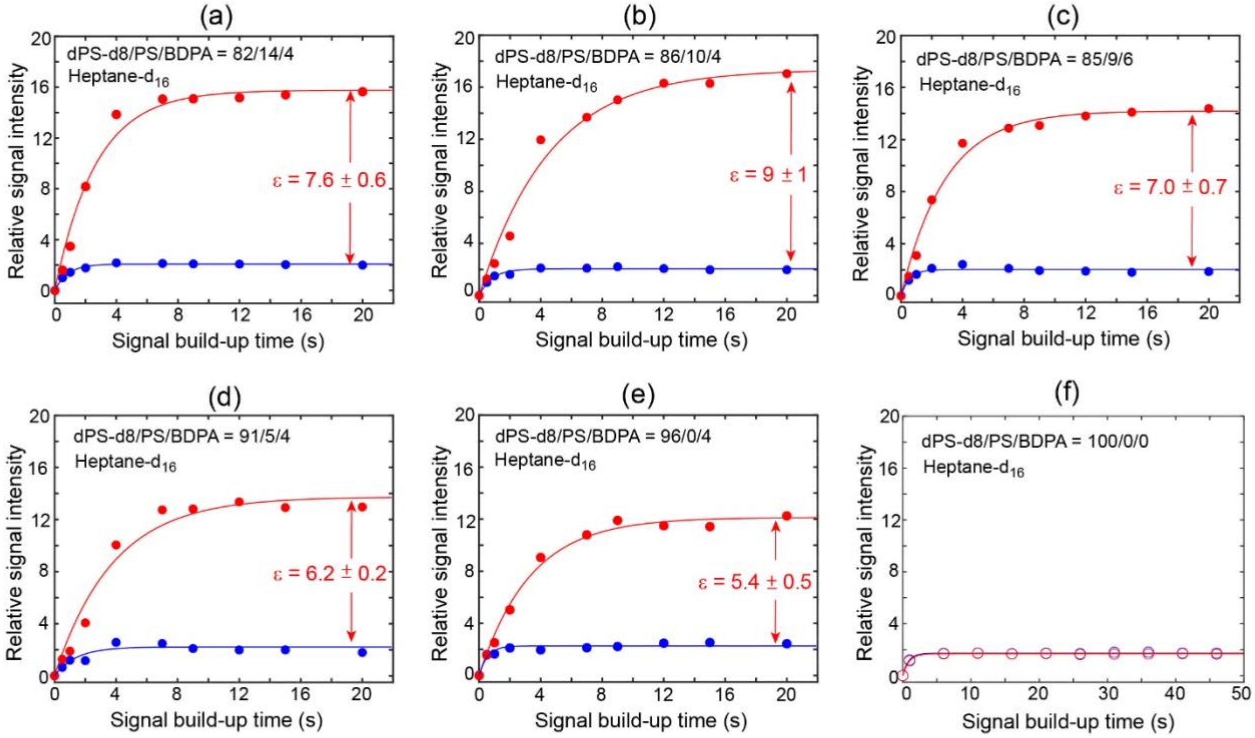

Figure S5. The observed ^1H DNP effect with varying the dPS/PS ratio (a-f) and different relative amounts of BDPA (b, c, and f). All experiments were conducted at 180 ± 5 K, introducing a thermal effect specific to temperature below 185 K (see below).

DNP enhancement increases with the degree of deuteration.[6,7] Our research thus involved additional ^{1}H DNP NMR experiments like those illustrated in Supplementary Figure S5. These involved varying the ratio of dPS/PS (a-f) and the relative amount of BDPA (b, c, and f). As in the main text, the red data in Figure S5 were recorded with the microwave turned on, while the blue data were recorded with the microwave turned off. The various parameters used to acquire the spectra are also the same as those described in the main text. Although the data are not shown here, during the initial pilot experiments, we used non-deuterated polystyrene and/or heptane solutions, resulting in no observed DNP phenomenon.

While keeping the total amount of BDPA fixed at 4%, Figure S5 examines the consequence of varying the amount of non-deuterated PS in small steps from 14 % (a) to 0% (e and f). In the case of the 0% PS condition, we utilized the residual ^{1}Hs present in dPS (98 atom % D). The highest DNP enhancement factor (ε = 9) was observed when the PS content was 10% (b), which is consistent with the results shown in Figure 2 of the main text. One point to note is that this experiment was conducted at a temperature of 180 ± 5 K, which entails a thermal heating effect (see section 8). The results shown in Figure S5c demonstrate the outcome when the BDPA content was increased to 6%, while maintaining the same dPS/PS ratio of 9/1 as in Figure S5b –which yielded the best result in terms of the dPS/PS ratio. With this higher BDPA content the ε value decreased, so best results were achieved with 4% BDPA. Solvent DNP was also absent if BDPA is not doped into the PS matrix as expected (f).

6. **Additional DNP-enhanced solution ^{1}H NMR examples.**

Figure S6 shows additional DNP-enhanced ^{1}H NMR spectra acquired under similar conditions as shown in Figures 2 and 3 of the main text, but on samples with slightly different compositions. One of those samples (panel a) used a dPS/PS ratio of 90/10 wt%, but with an increased amount of BDPA content, resulting in a overall composition of dPS/PS/BDPA = 84.9/9.4/5.7 w/w%; the other (panel b) contained only dPS (without PS) as polymer, comixed with 4% BDPA to yield a dPS/BDPA = 96/4 w/w% composition. Compared to the experiments introduced in Figures 2 and 3 of the main text, the

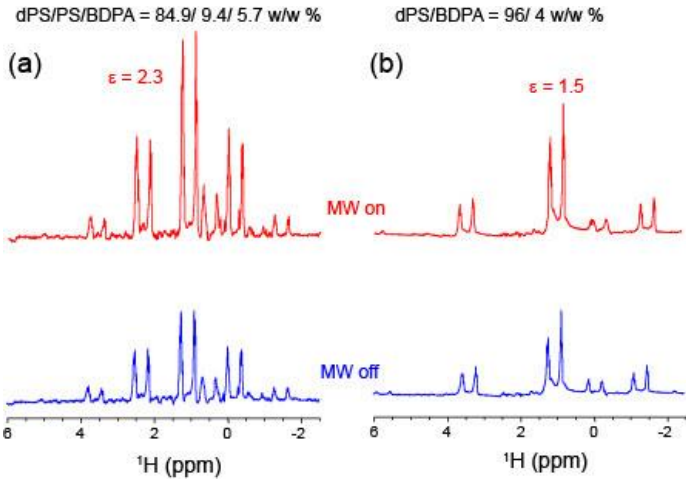

Figure S6. ^{1}H DNP spectra of the LMw samples dPS/PS/BDPA = 84.9/9.4/5.7 w/w % (a) and dPS/BDPA = 96.2/3.8 w/w % measured at 190 K. Spectra measured at this temperature are free from the thermal effect as shown in Figures 2 and 3 in the main text. The observed enhancement factor, e, were about 2.5 (a) and 1.5 (b), respectively.

observed ^{1}H DNP enhancement factors for these alternative sample compositions were lower: ε = 2.5 for (a) and ε = 1.5 for (b). Still, the overall appearances of the spectra in terms of excellent centerband resolution and numerous spinning sidebands, were similar in all cases.

7. The origin of spinning sidebands in liquid-state ^{1}H NMR.

All liquid-state ^{1}H NMR spectra measured in this DNP study exhibited spinning sidebands, even though the measurements are performed on an isotropic fluid. We ascribe this to the RF inhomogeneity present in the MAS DNP NMR probe used for obtaining the spectra. Indeed, it is known that both amplitude and phase RF inhomogeneities may be experienced by the sample at different positions within the coil. As the sample begins to rotate, spins experience this dependence, and it ends up modulating the signals they emit over their FIDs.[8] The same phenomenon is thus observed when using any MAS probe: Supplementary Figure S7a shows ^{1}H spectra of D_2O measured under static condition (bottom), spinning at 467 Hz (middle), and spinning at 2.7 kHz (top). As shown in these spectra, the line width of the ^{1}H spectrum recorded under spinning conditions is very narrow, with a full width at half height (FWHH) of approximately 1.5 Hz. Supplementary Figure S7b also presents spectra of the heptane-d_{16} sample recorded at room temperature under both spinning (ν_r = 417 Hz) and static conditions. In the spinning spectrum, peaks corresponding to CDH and CD_2H are observed at 1.4 ppm and 0.88 ppm, respectively. In contrast, all static spectra show ^{1}H signals as a broadened bump, even under optimal shimming conditions. Notice that all these samples had no PS or radical in them, and yet evidenced strong spinning sidebands and <1000 Hz spinning rates.

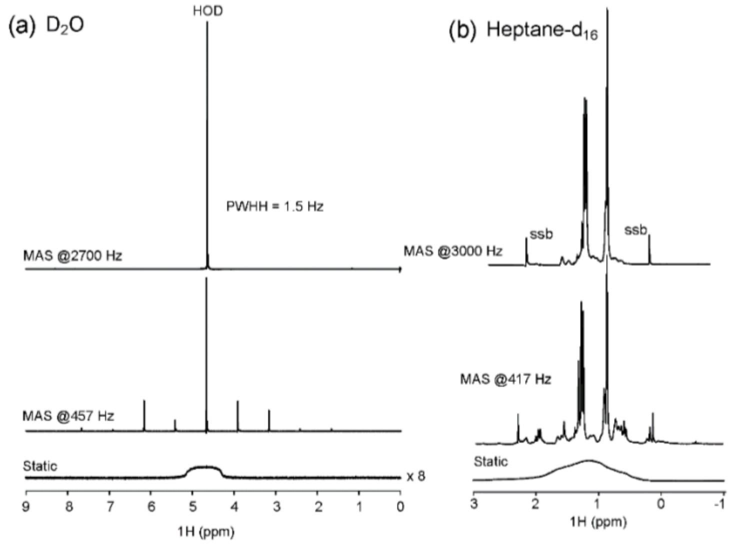

Figure S7. NMR spectra of deuterated D_2O (99.9 atom % D) and deuterated heptane-d_{16} (98 atom % D), acquired under both magic angle spinning (MAS) and static conditions using a 3.2 mm MAS probe. The measurements were performed on our DNP spectrometer at room temperature, with optimal shimming conditions.

8. Deriving the liquid-state PM-DNP buildup kinetics

This paragraph describes the kinetics of the particle-mediated liquid-state ^{1}H DNP experiment, as described by the two-step e$^-$ → ^{1}H-solid (PS) → ^{1}H-liquid (heptane) polarization transfer process. The first step is a typical DNP whereby electron polarization is transferred to the ^{1}H nuclei in the solid-state; the second step involves a crucial intermolecular cross-relaxation whereby electron polarization is ultimately transferred to the ^{1}H nuclei of liquid-state heptane. The time dependence of this latter process will be described by

$$\frac{dS}{dt} = -R_S(S - S_0) - \sigma_{SL}(L - L_0) \quad \text{(S1)}$$

$$\frac{dL}{dt} = -R_L(L - L_0) - \sigma_{LS}(S - S_0) \quad \text{(S2)}$$

where S and L denote the solid and liquid species respective magnetizations, R = 1/T_1 are their self-relaxation rate constants, S_0 and L_0 are their thermal equilibrium magnetizations, and the σs denote the cross-relaxation between species. Eqs. (S1) and (S2) will also describe the enhancements of these two proton reservoirs

$$\frac{d\varepsilon_S}{dt} = -R_S(\varepsilon_S - 1) - \sigma_{SL}\frac{L_0}{S_0}(\varepsilon_L - 1) \quad \text{(S3)}$$

$$\frac{d\varepsilon_L}{dt} = -R_L(\varepsilon_L - 1) - \sigma_{LS}\frac{S_0}{L_0}(\varepsilon_S - 1)\,, \quad \text{(S4)}$$

where

$$\varepsilon_S = \frac{S}{S_0},\ \varepsilon_L = \frac{L}{L_0}\,. \quad \text{(S5)}$$

To use these kinetics in order to describe PM-DNP, an additional term is needed to account for the DNP ^{1}H enhancement imparted by the electron. This can be done by adding into Eq. (S3)'s right-hand side an additional $B_S\left(\frac{P_e}{P_S} - \varepsilon_S\right)$ term describing the solids DNP. This, coupled to the fact that we are considering a homonuclear cross-relaxation process where $\frac{S_0}{L_0} = \frac{L_0}{S_0} = 1$ and together with Eq. (S4), leads to Eqs. (1) and (2) in the main text. To solve for the solution-state ^{1}H enhancement dynamics we use that, for all instances of interest, the term $\sigma_{SL}(\varepsilon_L - 1)$ in Eq. (S3) will be (in absolute value) much smaller than $R_S(\varepsilon_S - 1)$, and hence can be disregarded. Integration of Eq. (S3) then leads to

$$\varepsilon_S = \frac{B_S\varepsilon_S^0 + R_S}{(B_S + R_S)} + k \cdot exp[-(B_s + R_s)t]. \quad \text{(S6)}$$

As $\varepsilon_S = 0$ at t = 0,

$$k = -\frac{B_S\varepsilon_S^0 + R_S}{(B_S + R_S)} \quad \text{(S7)}$$

and Eq. (S6) becomes

$$\varepsilon_S = \frac{B_S\varepsilon_S^0+R_S}{(B_S+R_S)}\{1 - exp[-(B_s + R_s)t]\}. \tag{S8}$$

When the magnetization of the PS particles transfers to the heptane spins via NOE, an initial rate approximation can be used, whereby $\varepsilon_s(t = 0) = -\varepsilon_s^0$ –the negative sign arising due to the large size of the polarizing particles. Then, inserting Eq. (S8) into Eq. (S4), we will have

$$\frac{d\varepsilon_L}{dt} = -R_L\varepsilon_L + R_L + \sigma_{LS} + \sigma_{SL}\left(\frac{B_s\varepsilon_s^0 + R_s}{(B_s + R_s)}\right)$$

$$-\sigma_{LS}\left(\frac{B_s\varepsilon_S^0+R_S}{(B_s+R_S)}\right) exp[-(B_s + R_s)t]. \tag{S9}$$

To integrate Eq. (S9) we use the fact it has the form:

$$\frac{dy}{dt} = -ay + b + ce^{-gt} \tag{S10}$$

with y = ε_L, a = R_L, b = $R_L + \sigma_{LS} + \sigma_{SL}\left(\frac{B_S\varepsilon_S^0+R_S}{(B_S+R_S)}\right)$, c = $-\sigma_{LS}\left(\frac{B_S\varepsilon_S^0+R_S}{(B_S+R_S)}\right)$, and g = $(B_S + R_S)$.
Rearranging the terms in Eq. (S10) as:

$$\frac{dy}{dt} + ay = b + ce^{-gt} \tag{S11}$$

and multiplying both sides by e^{at}, we get:

$$e^{at}\frac{dy}{dt} + ae^{at}y = (b + ce^{-gt})e^{at} \tag{S12}$$

This equation can be simplified to:

$$\frac{d}{dt}(e^{at}y) = be^{at} + ce^{(a-g)t}. \tag{S13}$$

Integrating both sides then results in:

$$y = \frac{b}{a} + \frac{c}{a-g}e^{-gt} + k'e^{-at}. \tag{S14}$$

Since y$(t = 0)$ = $\varepsilon_L(t = 0) = 1$, we can determine the integration factor k' as:

$$k' = 1 - \frac{b}{a} - \frac{c}{a-g}. \tag{S15}$$

Using this yields the time dependence

$$y(t) = \frac{b}{a} + \frac{c}{a-g}e^{-gt} + (1 - \frac{b}{a} - \frac{c}{a-g})e^{-at} \quad . \tag{S16}$$

When all symbols in Eq. (S16) are replaced by the above-mentioned definitions, it yields Eq. (4) in the main text: the time-dependent magnetization transferred from the solid to the liquid, which was the kinetics we were aiming to obtain.

Figure S8 presents simulated ^{1}H DNP build-up curves, calculated based on Eq. S16 (corresponding to Eq. 4 in the main text). These simulations illustrate the expected changes in ^{1}H DNP build-up behavior when key parameters in the two-step e$^-$ → ^{1}H-solid (PS) → ^{1}H-liquid (heptane) PM-DNP mechanism, ε_S^0, $\mathrm{T_1(S), BS(S), T_1(L)}$, and σ_{SL}, are varied. Panels (a)–(e) show how the build-up curves respond to changes in: (a) ε_{ps}^0; (b) $\mathrm{T_1(S)}$; (c) Bs; (d) σ_{SL}; (e) $\mathrm{T_1(L)}$. Notice how increasing ε_S^0, $\mathrm{T_1(S)}$, $\mathrm{T_1(L)}$, Bs and σ_{SL} lead to sizable increases in the maximum ^{1}H DNP build-up curves, suggesting in turn the PM-DNP experiment has ample room to grow. And while manipulating some of these parameters like ε_S^0 might be hard, a lot of flexibility in the other parameters could arise by further chemical manipulations.

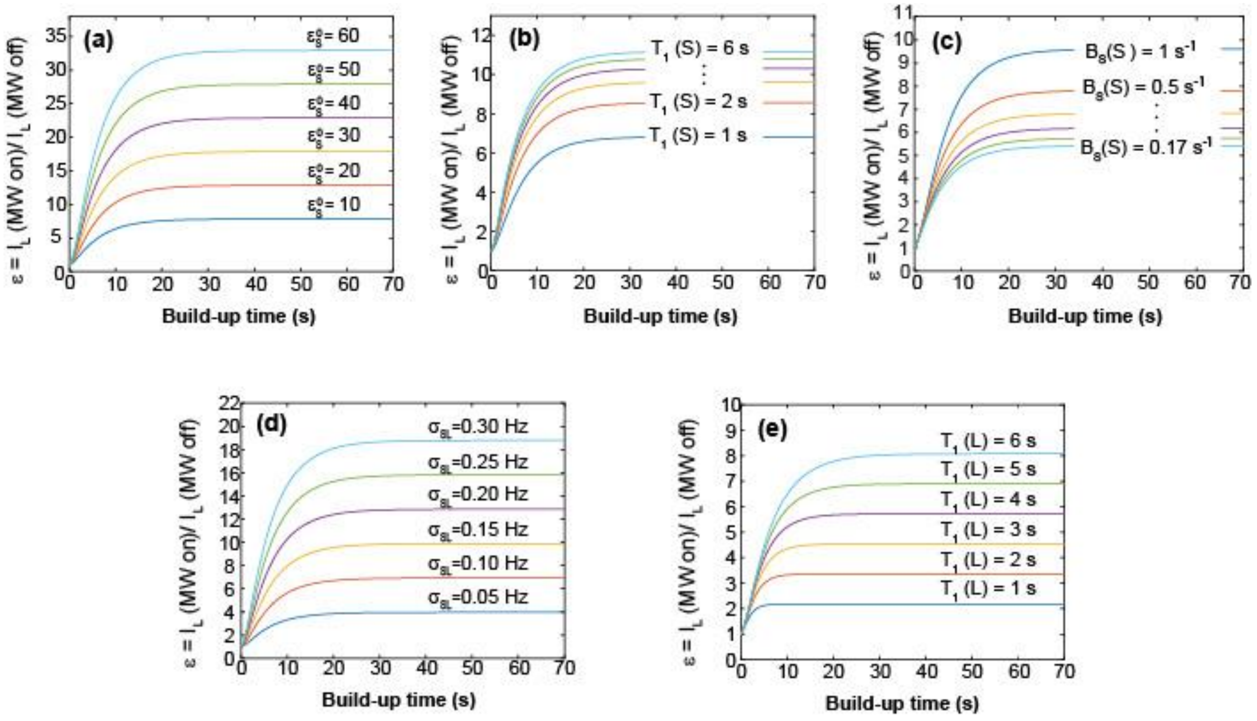

Figure S8. Simulated ^{1}H liquid state DNP build-up curves derived from Eq. (S16), upon varying the indicated. For each case, the parameters not under variation were kept constant as: ε_S^0 = 10, $\mathrm{T_1(S)}$ = 1.51 s, $\mathrm{B_S(S)}$ = 2.01 s^{-1}, $\mathrm{T_1(L)}$ = 5.8 s, and σ_{SL} = 0.2.

9. ^{1}H(PS)→^{1}H(Heptane) cross-relaxation rate measurements by saturation transfer difference (STD) experiments

To confirm that intermolecular polarization transfers from the PS particle surface to the ^{1}Hs of liquid heptane via cross-relaxation could serve as the logical mechanism underlying PM-DNP, a series of saturation transfer difference (STD) experiments were independently conducted to evaluate the particle→solvent cross-relaxation rates (Figure S9). The experiment was performed on a dPS/PS/BDPA powder sample (86.4/9.6/3.8) dispersed in heptane-d$_{16}$ at 190 K, using similar sample composition and temperature conditions as for the liquid-state ^{1}H PM-DNP –but without

microwave irradiation. The residual ^{1}H NMR spectrum of liquid heptane-d_{16} was recorded by applying a 90° pulse after selectively irradiating the ^{1}H peak of polystyrene at approximately 7 ppm for saturation using a train of Gaussian pulses; after comparing with a similar STD experiment but performed upfield of the pentane NMR resonances at -20 ppm. (Gaussian-5µs)$_n$ trains, where the number of pulses (n) was varied (n = 1, 2, 3...), where used, with watch Gaussian pulse lasting 30 ms, and involving 2 mW (90 Hz nutation field) powers. STD curves for the methylene (CDH) and methyl (CD_2H) groups were calculated as $(I_0 - I_n)/I_0$, where I_0 is the peak intensity without applying the saturation pulse train, and I_n is the peak intensity recorded after applying the saturation pulse train with n Gaussian pulses. The STD curves for the methylene (CDH) and methyl (CD_2H) moieties were plotted as a function of time (increasing n). These STD curves were fitted to A(1 - exp(-σt)) as a function of time, from which the σ value cross-relaxation rate values were obtained. These signal transfer rates σ_{SL} for the methylene (CDH) and methyl (CD_2H) groups were determined as 0.12 ± 0.06 s^{-1} and 0.13 ± 0.07 s^{-1}, respectively.

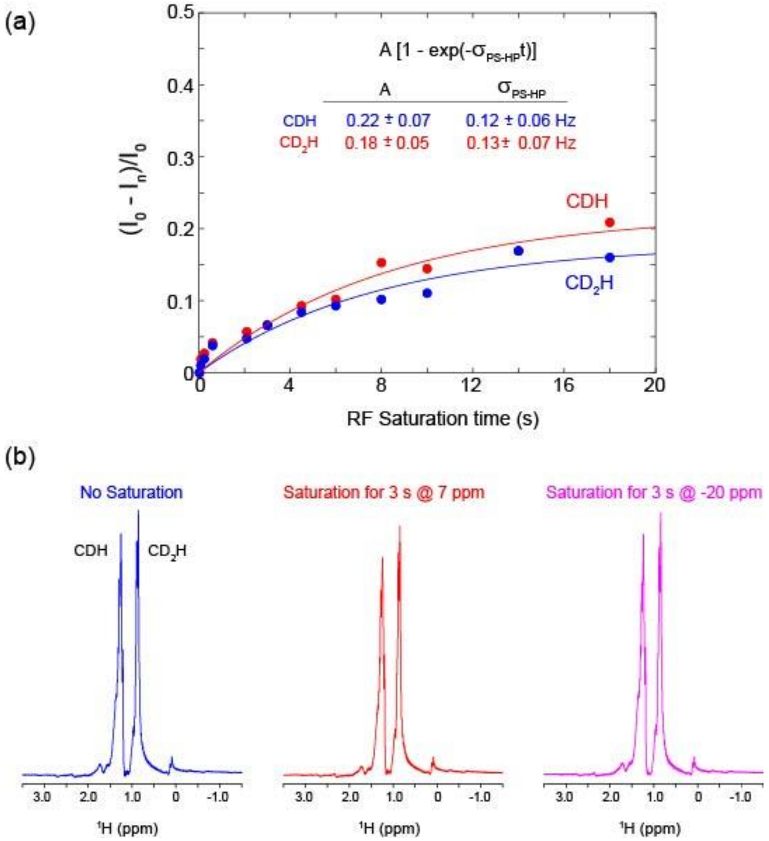

Figure S9. ^{1}H STD experiments performed on heptane-d_{16} with dispersed micro-powders of dPS/PS/BDPA (86.4/9.6/3.8) recorded at 190 K. (a) STD curves, $(I_0 - I_n)/I_0$, for the methylene (CDH) and methyl (CD_2H) groups plotted as a function of time. The inset table displays the best-fit parameters obtained by fitting the curves to the function A(1 - exp(-σt)), where A represents the amplitude of the STD intensity, and σ corresponds to the cross-relaxation rate. (b) Representative ^{1}H NMR spectra recorded under different conditions: without applying the saturation pulse (blue), after applying a 3 s saturation pulse (n = 100) at 7 ppm (red), and after applying the same saturation pulse sequence at an offset frequency of -20 ppm (pink). Notably, no saturation transfer effect was observed when the saturation pulse was applied at -20 ppm, confirming the specificity of the transfer mechanism.

10. ^{1}H T_1 relaxation of heptane-d_{16} at 190 K with and without dispersed dPS/PS microparticles.

^{1}H T_1 relaxation measurements were performed at 190 K for pure heptane-d_{16} (a) and heptane-d_{16} with dispersed dPS/PS/BDPA (86.5/9.6/3.9 wt%) (b). Notably, the T_1 relaxation times of the two samples differ significantly. In pure heptane-d16, the T_1 values for both the methylene and methyl moieties are 0.94 ± 0.04 s. In contrast, for heptane-d_{16} with dispersed dPS/PS/BDPA microparticles, the T_1 values not only vary between the methylene and methyl groups but are also substantially longer, measuring 4.5 ± 0.4 s and 7.6 ± 0.9 s, respectively. The increase in T_1 values in heptane-d_{16} with dispersed microparticles is primarily due to the increased local viscosity slowing down molecular motion and restricted molecular motion near the particle surface. These effects shift the system to the optimal cross-relaxation regime between ^{1}H (PS) on the particle surface and ^{1}H (HP) in the liquid phase.

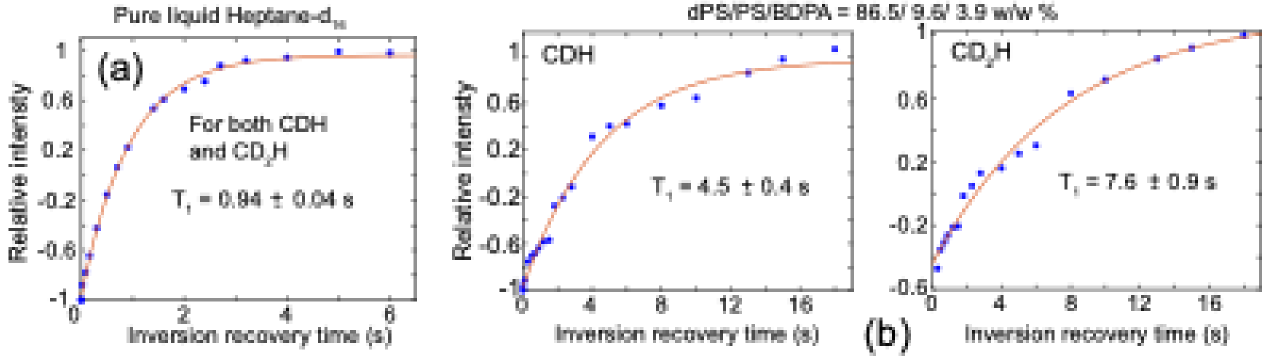

Figure S10. ^{1}H T_1 measurements carried out at 190K on (a) pure heptane-d_{16} and (b) heptane-d_{16} with dispersed dPS/PS/BDPA.

11. Temperature-dependent On- and Off-Overhauser microwave irradiation

As shown in Figure 2 of the main text, the frequency-dependent ^{1}H NMR sweep profile enables precise identification of the optimal Overhauser Effect (OE) frequency at 600.65 MHz. Additionally, at frequencies outside the OE condition, signal enhancement due to thermal heating was negligible under the nominal experimental temperature conditions (185~ 195 K). The supplementary data shown in Figure S11 presents additional experimental results designed to clearly distinguish between the DNP effect (a-c) and signal enhancement caused purely by thermal effects (d-f), by varying the temperature at selected frequencies representing both the OE position (600.65 MHz) and an off-OE position (600.35 MHz). Three differently calibrated temperatures were used in the experiments: 180 ± 5 K (a and d), 185 ± 5 K (b and e), and 220 ± 5 K (c and f). The reason for the error of approximately ±5 K has been explained above section. The 180 K condition is close to the freezing point of heptane (182 K), although the actual freezing temperature would be lower due to the dispersion of BDPA-doped dPS/PS particles. Under the OE condition, the 180 K case exhibited the best enhancement ($\varepsilon = 9$), despite being close to the freezing point of

heptane, which results in broadened NMR lines and introduces an additional thermal effect (a). The presence of dispersed BDPA/dPS/PS particles ensures that the solution remains in a liquid state. The temperature of 185 K still exhibits the desired effect with ε = 5.5 while remaining sufficiently above the freezing point (b). The third temperature, 220 K, is too high, resulting in the absence of the desired DNP effect (c). In the case of experiments conducted at the off-OE frequency of 600.35 MHz (d-f), no signal enhancement should be observed at any temperature when the microwave is turned on. However, as shown in Figure (d), a non-negligible signal increase (ε = 3.7) is observed, which is attributed to the thermal heating effect. However, as expected, at the higher temperature of 185 K, no signal enhancement due to thermal heating was observed. This case is similar to the results shown in Figure 2 of the main text. It is noteworthy that in the results from the 180 K experiment, when accounting for the thermal heating effect, the adjusted ε value of 9 - 3.7 = 5.3 is like the results observed at 185 K in (b).

The off-OE changes observed at 180K, deserve further comment. Although not shown here, additional non-DNP variable-temperature MAS NMR experiments conducted on other ^{1}H 600 MHz spectrometers on PS-containing heptane-d_{16}, octane-d_{18}, and dodecane-d_{24} solutions, indicate that the primary reason for the observed off-OE changes shown in Fig. S8 at 180 K, arise due to changes in the molecular mobility of the solvents near their freezing point, leading to enhanced NMR T_2 relaxation. When the microwave is turned on, a small heating effect likely increases molecular mobility and/or reduces the viscosity of the heptane, leading to an increase in T_2

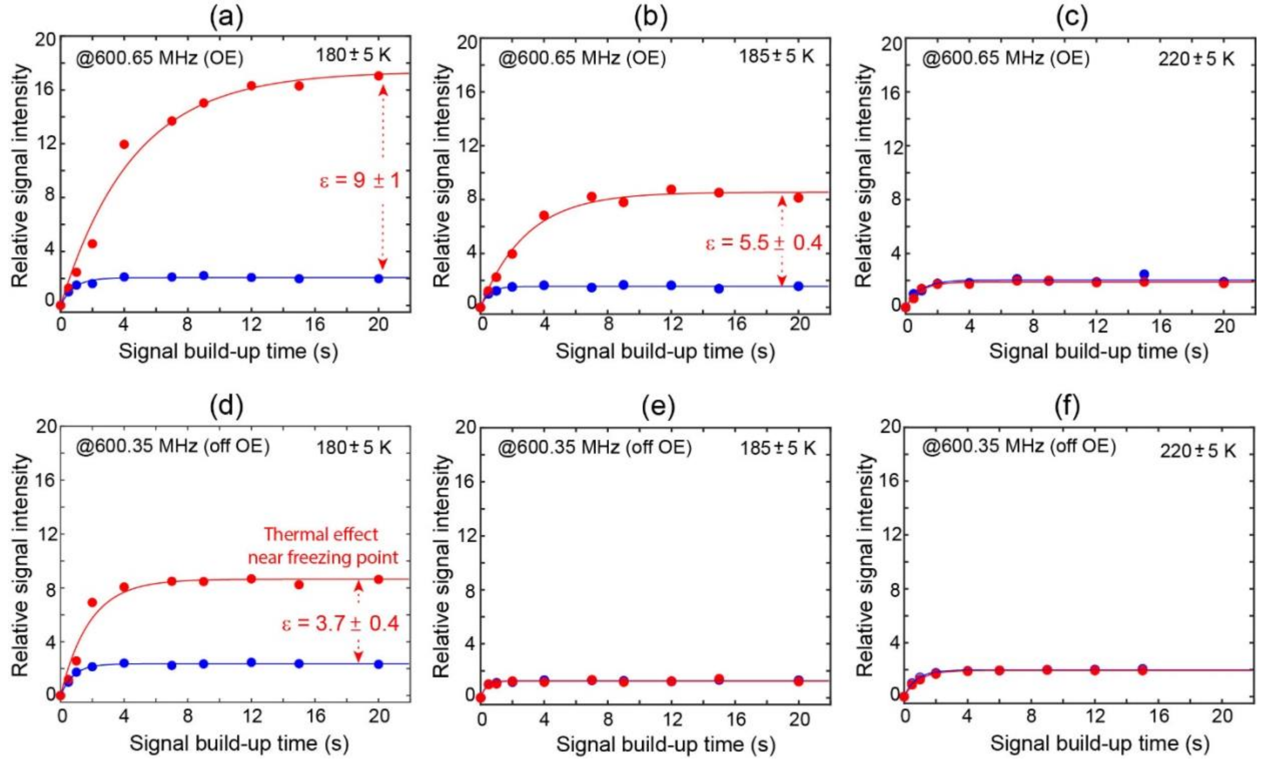

Figure S11. Microwave-on (red) and -off (blue) build-up curves as a function of temperature at the on-OE (600.65 MHz) and a selected off-OE frequency (600.35 MHz).

relaxation time; under the Hahn echo conditions we used (delay time = 250 μs), it is likely that part of a broad peak in the microwave-off spectrum was lost during the refocusing, resulting in an underestimation of the microwaves-off spectral intensity. At temperatures above 185 K, however, even the microwave-off spectral lines become so narrow, that such errors are avoided, and these apparent thermal heating effects disappear.

References:

1. J. van Tol, L. Brunel and R. Wylde, "A quasi-optical transient electron spin resonance spectrometer operating at 120 and 240 GHz," *Review of Scientific Instruments,,* vol. 76, no. 7, pp. 074101-1, 2005.
2. N. S. Dalal, D. E. Kennedy and C. A. McDowell. "EPR and ENDOR studies of hyperfine interactions in solutions of stable organic free radicals." *J. Chem. Phy.*, vol. 61, no. 5, 1689–1697, 1974.
3. D. Goldfarb, B. Epel, H. Zimmermann, and G. Jeschke, "2D TRIPLE in orientationally disordered samples—a means to resolve and determine relative orientation of hyperfine tensors." *J. Magn. Reson.* 168, 75–87 (2004).
4. M. L. Huber, I. M. Abdulagatov and R. A. Perkins, "Reference Correlation of the Viscosity of n-Heptane from the Triple Point," *J. Phys. Chem. Ref. Data,* vol. 43, no. 2, p. 023103, 2014.
5. K. R. Thurber and R. Tycko, "Measurement of sample temperatures under magic-angle spinning from the chemical shift and spin-lattice relaxation rate of 79Br in KBr powder," J. Magn. Reson., vol. 196, no. 1, pp. 84-87, 2009.
6. T. Maly, G. T. Debelouchina, V. S. Bajaj, K. N. Hu, C. G. Joo, M. L. Mak-Jurkauskas, J. R. Sirigiri, P. C. A. Van Der Wel, J. Herzfeld, R. J. Temkin and R. G. Griffin, "Dynamic Nuclear Polarization at High Magnetic Fields," *J. Chem. Phys.,* vol. 128, p. 052211, 2008.
7. T. V. Can, M. A. Caporini, F. Mentink-Vigier, B. Corzilius, J. J. Walish, M. Rosay, W. E. Maas, M. Baldus, S. Vega, T. M. Swager and R. G. Griffin, "Overhauser Effects in Insulating Solids," *J. Chem. Phys.,* vol. 141, p. 064202, 2014.
8. R. Avni, O. Mangoubi, R. Bhattacharyya, H. Degani and L. Frydman, "Magnetization transfer magic-angle-spinning z-spectroscopy of excised tissues," *J Magn Reson.,* vol. 199, no. 1, pp. 1-9, 2009.
